# Development and Evaluation of a Home Enteral Nutrition Team

**DOI:** 10.3390/nu7031607

**Published:** 2015-03-05

**Authors:** Sarah Dinenage, Morwenna Gower, Joanna Van Wyk, Anne Blamey, Karen Ashbolt, Michelle Sutcliffe, Sue M. Green

**Affiliations:** 1 University Hospital Southampton NHS Foundation Trust, Southampton, Hampshire, SO16 6YD, UK; E-Mails: sarah.dinenage@nhs.net (S.D.); anne.blamey@uhs.nhs.uk (A.B.); Karen.ashbolt@uhs.nhs.uk (K.A.); michelle.sutcliffe@nhs.net (M.S.); 2 Solent NHS Trust, Adelaide Health Centre, Western Community Hospital Campus, Southampton, SO16 4XE, UK; E-Mail: morwenna.gower@solent.nhs.uk; 3 Nutricia Ltd., Trowbridge, Wiltshire, BA14 0XQ, UK; E-Mail: Johanna.vanwyk@nutricia.com; 4 Solent NHS Trust/University of Southampton, Highfield, Southampton, SO17 1BJ, UK

**Keywords:** Enteral tube feeding, home enteral nutrition, primary care

## Abstract

The organisation of services to support the increasing number of people receiving enteral tube feeding (ETF) at home varies across regions. There is evidence that multi-disciplinary primary care teams focussed on home enteral nutrition (HEN) can provide cost-effective care. This paper describes the development and evaluation of a HEN Team in one UK city. A HEN Team comprising dietetians, nurses and a speech and language therapist was developed with the aim of delivering a quality service for people with gastrostomy tubes living at home. Team objectives were set and an underpinning framework of organisation developed including a care pathway and a schedule of training. Impact on patient outcomes was assessed in a pre-post test evaluation design. Patients and carers reported improved support in managing their ETF. Cost savings were realised through: (1) prevention of hospital admission and related transport for ETF related issues; (2) effective management and reduction of waste of feed and thickener; (3) balloon gastrostomy tube replacement by the HEN Team in the patient’s home, and optimisation of nutritional status. This service evaluation demonstrated that the establishment of a dedicated multi-professional HEN Team focussed on achievement of key objectives improved patient experience and, although calculation of cost savings were estimates, provided evidence of cost-effectiveness.

## 1. Introduction

The number of people receiving food and fluid intake via a gastrostomy tube in primary care has increased [1,2] and it is now a relatively common intervention in the UK [3]. Poorly managed gastrostomy tubes and enteral feeding in the community setting can lead to complications, hospital admission [3] and dissatisfaction with care provision [4]. Other aspects of poor management include wastage of feeds and equipment. Therefore, it is important that people with gastrostomy tubes, their carers and primary care services are supported to manage the therapy effectively.

Management of a gastrostomy tube at home requires development of knowledge and skills and life style adaptations. People with a gastrostomy tube report it to be a burden, time-consuming and disruptive to their lives [5,6]. Further, relatives of people living at home with a feeding tube have described managing the new life situation it presents as a struggle [4]. Appropriate education, training and support is required both to ensure a smooth transition between care settings and safe and effective management within the primary care setting [7,8]. UK NICE guidelines [9] outline that people receiving enteral tube feeding (ETF) in the community should “be supported by a coordinated multidisciplinary team”. There are a number of ways services can be organised to support people with home enteral feeding (HEF) [10] and a range of intervention strategies have been described [11]. A recent systematic review highlighted that a standardised care coordination model with a multidisciplinary team can improve patient outcomes and reduce health care costs, but there is insufficient evidence to determine the effectiveness of a particular intervention or team composition [11]. Outpatient-based services, such as enteral nutrition support clinics, have been suggested to improve care quality and reduce hospital readmissions, tube-related complications and costs [12]. In some areas, teams based in community settings (often termed Home Enteral Nutrition (HEN) teams) have been formed to provide a service for patients in their own homes [13] and these have been associated with improved care outcomes. For example, Klek *et al.* [14] report cost savings following the introduction of commercial formulas and guidance from a nutrition support team. In the UK, Kurian *et al.* [3] suggested that the direct actions of a HEN Team comprising dietitians and assistants in one UK city potentially resulted in the avoidance of 227 hospital admissions over a year. This paper aims to describe the development, interventions and evaluation of a multidisplinary HEN Team in one city in England.

## 2. Materials and Methods

### 2.1. Team Development

Local funding was secured for six months to form a HEN Team and scope the potential for improved patient care and cost savings. Following this, a further 12 months of funding totaling £84,071 was awarded, which allowed the service to be embedded in local healthcare provision. The Team comprised a dietitian, a speech and language therapist, a homecare company nurse and a nutrition nurse. All Team members worked part-time with the Team whilst holding other professional roles within their contracted time.

### 2.2. Team Aim and Objectives

The aim of the HEN Team was to provide a service for people over the age of 18 with gastrostomy tubes in a UK city (population approximately 265,000) focussed on improving patient experience and quality of nutritional care. The key objectives for the Team and how they were achieved are outlined below to give information on the interventions and how they were implemented.

#### 2.2.1. Objective: Development of a Care Pathway and Links with Other Services

**Figure 1:**
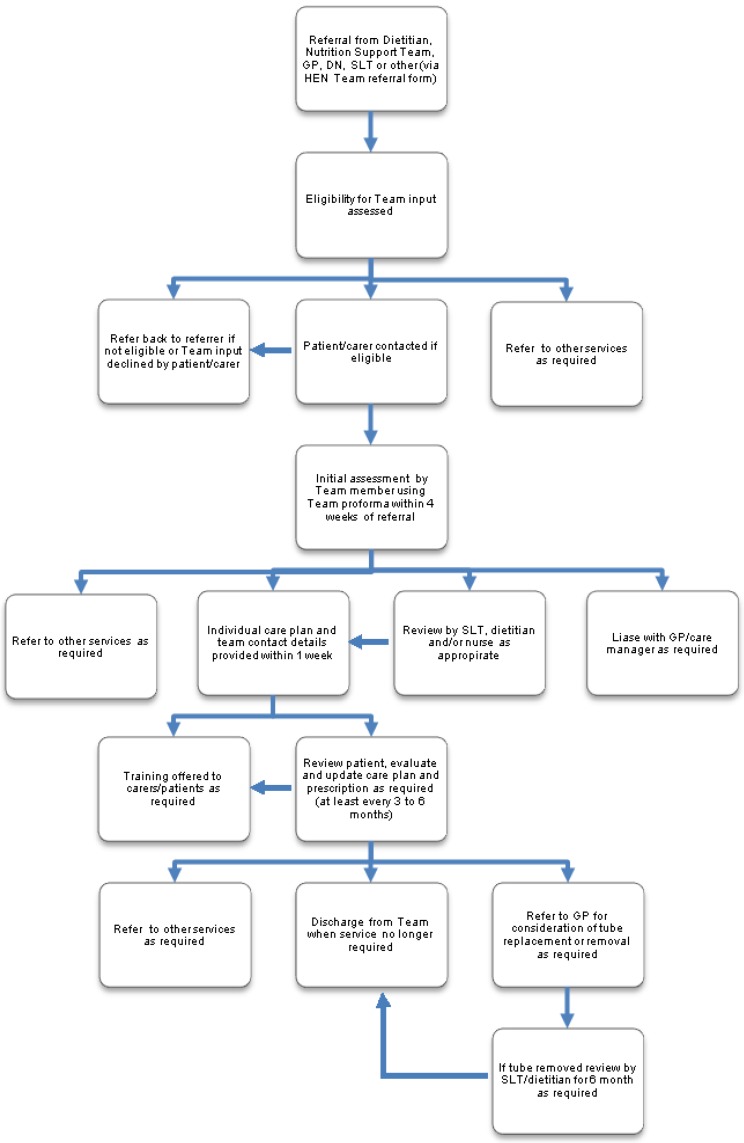
Care pathway for management of patients with enteral tubes by Home Enteral Nutrition (HEN) Team, Key: GP: General practitioner, DN: district nurse, SLT: Speech and language therapist.

An integrated care pathway in the form of a flow chart was developed and embedded into practice (Figure 1). The pathway outlined the stages of management for people concerning their feeding tubes, including how referrals were created, timeframes for assessment and review, and discharge from the service. The roles and responsibilities of each Team member were also outlined. The pathway provided a framework for activity and acted as a point of reference. Communication with other teams (such as the acute dietetic service and the local Nutrition Support Team) involved in caring for people with enteral feeding tubes enabled the development of professional networks which facilitated and improved coordination of care.

#### 2.2.2. Objective: Care Provision with Reference to NICE Guidelines [8,9]

The Team provided a service delivering multidisciplinary care concerning nutrition for adults with gastrostomy tubes in the home setting. The following activities were undertaken with each patient:

- Review of use of feed ancillaries (giving sets, syringes, connectors, replacement parts), feed, thickener prescription and adjustment of plan of care if required on admission to the service and at least 6 monthly subsequently
- Review of stoma and tube and adjustment of plan of care if required
- Repair of tube as required
- Planned and emergency balloon-retained gastrostomy tube replacement at home where not clinically contraindicated
- Review of type of balloon-retained gastrostomy tubes and change to longer-lasting tubes requiring fewer balloon volume checks where possible
- Review of route and preparation of medication and suggestions for change if appropriate

An individualised care plan for gastrostomy and nutritional management was developed for each patient. As well as providing support for patients and carers, the Team worked to reduce risks through the early identification of problems.

#### 2.2.3. Objective: Provision of Specialist Advice, Training and Education

The Team acted as a source of specialist advice on home ETF for patients, carers and other community practitioners. Members of the Team could be contacted by telephone during office hours. The Homecare Company continued to provide a 24 h helpline as part of their service contract. Relevant training and education was delivered to patients, carers, family members and nursing home staff in the form of in-house training and a study day.

#### 2.2.4. Service Evaluation

A pre-post evaluation of the impact of the presence of the HEN Team on selected patient outcomes derived from the HEN Team objectives outlined above was undertaken. Outcomes (except those related to hospital admission) for all 70 patients in the caseload were compared at one time point in the 12 months pre-HEN Team development (2011) and one time point following the first three months of the Team development (2012) or at the point of discharge from the Team. From this, the mean cost per day for each patient was calculated.

Hospital admission costs were calculated for 28 patients. This convenience sample was selected on the basis of known hospital admission. Sample size was determined by the time available to extract data from the hospital medical records recorded over a 24-month period. For these 28 patients, the mean cost per year of hospital admission was calculated from the review of 24 months of medical records pre-HEN Team development. These were then compared with the mean cost for 2012.

#### 2.2.5. Outcomes Included

- Number of patients whose risk of malnutrition decreased (measured using the Malnutrition Universal Screening Tool (MUST) [15])
- Number of patients, carers, healthcare staff and nursing home trained in gastrostomy tube care
- Number of patients who had tubes removed
- Estimates of cost of:
  ○ enteral feed prescription
  ○ thickening agents for dysphagia
- Frequency and length of hospital admission and hospital transport costs for ETF related problems for 28 patients in the caseload.

Data was obtained from hospital medical, dietetic, and community and homecare nursing records. A patient satisfaction survey in the form of a written questionnaire was undertaken at one time point during the 12 months. The survey comprised a 15-item anonymous postal questionnaire which was adapted from an existing service evaluation. The two items reported here concern the questions “Before your first appointment, did you know what to expect from the HEN Team?” (yes or no) and “How they would you rate the overall service received? (very poor, poor, OK, good or excellent).

This was distributed to all patients in the caseload by post with a stamped addressed envelope for return.

### 2.3. Statistical Analysis

SPSS 17.0 was used to record and analyse data using descriptive statistics and frequencies. Actual cost savings were estimated. Values for bed days were calculated using the following estimates derived from service managers and a Trust Finance Department: day case £250, general medical ward £177, acute medical unit (AMU) £240, critical care £1100, specialist rehabilitation unit £470. If the admission location was unclear, costs were calculated as 2 days on AMU and the remainder on a general medical ward. The cost of patient transport relating to hospital admissions was calculated using an average price of £55.77 per trip. The cost of feed and thickener for each patient per day was calculated and then an estimated cost per year obtained for each patient. These were then summed to produce an annual cost. Products were costed using the invoice supplied by the provider or from the current British National Formulary. Costs of gastrostomy tubes were obtained from manufacturers. Scheduled time in the radiology department was estimated at £250 per case.

### 2.4. Ethics and Governance

The study was registered as a service evaluation with the Trust and deemed not to require ethical committee approval. Confidentiality was maintained during the recording and analysis of the data. Personal identifying information was not recorded and patients were allocated a unique anonymous identification number.

## 3. Results

### 3.1. Caseload Characteristics

People from the age of 18 years and older were admitted to the caseload, although the mean age was 61 years (range 19 to 90 years). The majority of patients were female (60%). Nearly 70% lived in their own homes with the remainder living in nursing homes. A high proportion of patients (approximately 70%) had a percutaneously inserted gastrostomy tube. The remainder had a balloon retained gastrostomy (about 20%), “low-profile” gastrostomy or jejunostomy. The most frequently reported medical condition causing the need for a tube was cerebrovascular accident (about 25%) but other conditions that featured frequently in the population were neurological diseases and learning disability [16].

The HEN Team made a total of 595 contacts (face to face and via telephone) over the course of 2012, equating to a mean of 50 contacts per month or 8.5 contacts per patient over the year. A breakdown of contacts per discipline is shown in Table 1.

**Table 1:** Contacts by discipline.

	Dietitian	Speech and Language Therapist	Nurse	TOTAL
Number of contacts	241	187	167	595

### 3.2. Must Score

The proportion of patients at medium or high risk of malnutrition (MUST score greater than 0) was reduced from 41% to 25% suggesting reduced risk of malnutrition.

### 3.3. Training

Each patient, and where appropriate their carers, received training at the point of care. A study day entitled “Caring for tube feeds in the community” was held by the HEN Team and attended by 20 healthcare professionals and carers during the intervention period. The study day was not available in the pre-intervention period.

### 3.4. Number of Patients Who Had Tubes Removed

Over the course of the intervention period (2012) eight patients had their tubes removed, which is broadly in line with other published reports [3].

### 3.5. Estimated Cost Savings

Table 2 shows estimates of the cost savings for specific components of the care provision pre and post intervention.

Review of each patient’s enteral feed prescription by the dietitian, to ensure patient nutritional needs were being met, resulted in cost savings because some feeds were reduced or changed to standard feeds and bolus feeds (Table 2). Review of water and feed ancillaries for each patient resulted in some adjustments made to their equipment supply to ensure unnecessary components were not ordered and supplies were not excessive. For example, one patient was initially receiving two 500 mL bags of a concentrated feed with a two-pack connector. Following dietetic assessment, the patient was changed over to one bag of a high-energy feed, thus removing the need for the two-pack connector as well as reducing the number of bags of feed. This prescription was more appropriate for the patient as it was less labour-intensive. The cost savings as a result of review of water and feed ancillaries are not included in the calucation of the cost savings.

**Table 2:** Estimated costs pre and post introduction of the Home Enteral Nutrition Team (*n* = 70).

Component of provision	Calculation of estimated costs	Pre introduction estimated cost (2011)	Post introduction estimated cost (2012)	Estimated Cost differential
Enteral feed prescription per year	2011: mean cost of feed £9.18 per patient per day 2012: mean cost of feed £7.41 per patient per day	£234,505	£189,326	£45,179
Thickening agents for dysphagia	2011: daily thickener costs per patient £0.53 2012: daily thickener costs per patient £0.48	£13,542	£12,264	£1,278

Review of thickening agent use and type by the speech and language therapist resulted in cost savings as swallow ability of some patients improved (Table 2).

There were fewer hospital admissions for tube-related problems recorded in 2012 compared to 2010/2011 (Table 3). There were also fewer hospital admissions for routine balloon gastrostomy tube replacement. Over the course of 2012, the Team undertook tube replacement in the home for fourteen patients. Prior to the introduction of the HEN Team, some patients were attending radiology to have their balloon gastrostomy tube replaced. Manufacturers guidelines for the type of tube routinely used within the local area suggest three monthly replacements, so this potentially saved four radiology appointments yearly per patient. Additionally, over the course of 2012, seven patients were changed to a tube type which requires checking of the balloon volume and changing less frequently than the previous tube routinely used. This resulted in a reduction in tube cost of £93 per patient or £651 for the year. Cost of nurse visits to change the balloon volume were not calculated.

### 3.6. Patient Satisfaction Survey

Fourteen people returned the patient satisfaction survey (30% response rate). All respondents stated that they were were extremely satisfied with the services provided by the HEN Team, with 100% of respondents rating the overall service received as good or excellent. Over half the respondents to the survey indicated that they had not known what to expect from the HEN Team before the first visit, suggesting that the role of the HEN Team was unclear.

**Table 3:** Estimated costs of hospital admission pre and post introduction of the Home Enteral Nutrition Team (*n* = 28).

Costs component	Calculation of estimated saving	Pre-introduction estimated cost (mean per year)	Post-introduction estimated cost (2012)	Estimated cost differential
Hospital admission (frequency and bed days) for balloon gastrostomy tube replacement and tube related problems	2010/2011: 25 admissions 740 bed days 10 day cases 2012: 7 admissions 98 bed days 2 day cases	£82,943	£18,602	£64,341
Hospital transport for balloon gastrostomy tube replacement in hospital and tube related problems	2010/2011: Transport based on 25 admissions and 10 day cases 2012: Transport based on 7 hospital admissions and 2 day cases	£976	£502	£474

## 4. Discussion

Increasingly, community services are required to support people with ETF [17]. It is important to consider how a service can be provided that effectively meets the needs of patients, as currently there is little guidance on who should be included in the Team, the implementation process or evaluation of effectiveness [11]. The introduction of a new team to deliver a service previously not available in the local area was approached by forming team aims and objectives linked with clear measurable outcomes. This enabled care provision concerning ETF via gastrostomy to align closely with national guidelines [8,9] and expected standards of care [18]. The service evaluation measured by the outcomes generated from the team objectives indicated that the Team was able to provide a cost-effective community service, which improved the experience and quality of care of people with gastrostomy tubes. For a cohort of 70 patients, the introduction of a HEN Team was associated with crude estimated cost savings of £111,272 over one year. The service cost £84,071 to deliver, giving rise to an estimated net saving of £27,201 to the NHS.

Previous evaluations of similar teams have demonstrated greater cost savings [3]. Other activities of the HEN Team linked to the Team objectives potentially generated further costs savings but were not included in the cost savings analysis presented in this paper. These included review of ancillaries and water ordered for each patient, review of medications and transfer to oral medication with improved swallow, reduction in dressing use as a result of improved training, reduction in district nursing time for balloon volume changes, and recommendation for removal of gastrostomy tubes where swallow function improved sufficiently to enable adequate oral intake to maintain health. Calculating cost savings was a challenge for the Team, as it was an activity that none of the members had previously undertaken. The estimates of savings were crude, being based on figures that were estimates of cost. Nevertheless, some cost savings were clearly made. Other reported evaluations of teams have focussed on measurement of patient-reported outcome measures to demonstrate clinical effectiveness [19]; however, we considered it essential to demonstrate cost effectiveness in addition to patient satisfaction. A recent systematic review identified that evaluation of cost effectiveness of nursing practice is limited [20] and there is a need to consider cost implications in service delivery within community settings.

The multidisciplinary nature of the Team enabled rapid referral to other disciplines, consistent advice, more regular review, timely and appropriate clinical care and better provision of training. The speech and language therapist was a key member of the Team. She supported and empowered patients with dysphagia to comply with clinical recommendations concerning food and fluid intake. In addition, as each patient’s ability to swallow was reviewed regularly, any patient with the potential for improved swallow received ongoing speech and language therapy at home. This potentially reduced the risk of aspiration pneumonia and, for some patients, resulted in timely removal of their gastrostomy tube. This has a significant impact on patient experience. The psychosocial benefits of receiving oral intake should not be underestimated.

### 4.1. Limitations

The time since insertion of the tube was not taken into account in calculation of the cost savings. This is a limitation of this study as it has been suggested that there is a substantial risk of complications in the first few months of placement [21]. A design that allowed for a control group would have been preferable but was not feasible as a result of service requirements. Calculated cost savings were crude estimates due to the clinical Team’s inexperience in undertaking robust economic evaluation. However, it was considered important to attempt to demonstrate the value for the service to the local NHS economy, and demonstrates the need for clinicians to develop skills in evaluation of cost effectiveness of services. The pre-post intervention evaluation design did not allow for temporal variations in care environment to be evaluated, and the changes seen may have occurred without the introduction of the Team.

### 4.2. Practice Recommendations

A HEN Team has the potential to increase patient satisfaction and reduce costs associated with ETF in the community. The process of team development requires the setting of clear aims and key objectives focussed on enhancing patient experience. When implementing a specialist community healthcare service, it is important to consider how the service can be evaluated robustly so patient satisfaction and cost-effectiveness can be demonstrated.

## 5. Conclusion

ETF is a costly therapy, and a dedicated HEN Team can help to minimise the significant costs related to tube feeding and enhance the experience of people receiving tube feeding at home. These findings are consistent with other similar studies [11]. Through effective management and care delivery, it was shown that patients supported by the HEN Team were admitted to hospital less frequently, used less hospital transport and had reduced costs for feed and thickener. The reduction in hospital admissions is likely to be due to risk management by the HEN Team for complications such as tube blockage and deterioration. However, as the study design was pre- and post-evaluation, hospital admission may have been reduced as a function of time. Other components of the provision not measured would have been likely to contribute to costs savings.

## References

[1] Russell C.A., Rollins H. (2002). The needs of patients requiring home enteral tube feeding. Prof. Nurs..

[2] Smith T., British Association of Enteral and Parenteral Nutrition (2011). Annual BANS Report.

[3] Kurien M., White S., Simpson G., Grant J., Sanders D.S., McAlindon M.E. (2012). Managing patients with gastrostomy tubes in the community: Can a dedicated enteral feed dietetic service reduce hospital readmissions?. Eur. J. Clin. Nutr..

[4] Bjuresäter K., Larsson M., Athlin E. (2012). Struggling in an inescapable life situation: Being a close relative of a person dependent on home enteral tube feeding. J. Clin. Nurs..

[5] Jordan S., Philpin S., Warring J., Cheung W.Y., Williams J. (2006). Percutaneous endoscopic gastrostomies: the burden of treatment from a patient perspective. J. Adv. Nurs..

[6] Martin L., Blomberg J., Lagergren P. (2012). Patients’ perspectives of living with a percutaneous endoscopic gastrostomy (PEG). BMC Gastroenterol..

[7] National Institute for Health and Clinical Excellence (2012). Infection: Prevention and control of healthcare-associated infections in primary and community care.

[8] National Institute for Health and Clinical Excellence (2012). Quality standard for nutrition support in adults.

[9] National Institute for Health and Clinical Excellence (2006). Nutritional Support in Adults: Oral Nutrition Support, Enteral Tube Feeding.

[10] Green S., Dinenage S., Gower M., Van Wyk J. (2013). Home enteral nutrition: Organisation of services. Nurs. Older People.

[11] Majka A.J., Wang Z., Schmitz K.R., Niesen C.R., Larsen R.A., Kinsey G.C., Murad A.L., Prokop L.J., Murad M.H. (2014). Care coordination to enhance management of long-term enteral tube feeding: A systematic review and meta-analysis. J. Parenter. Enter. Nutr..

[12] Hall B.T., Englehart M.S., Blaseg K., Wessel K., Stawicki S.P., Evans D.C. (2014). Implementation of a dietitian-led enteral nutrition support clinic results in quality improvement, reduced readmissions, and cost savings. Nutr. Clin. Prac..

[13] Omorogieva O., Patel I. (2012). Home enteral nutrition and team working. J. Community Nurs..

[14] Klek S., Szybinski P., Sierzega M., Szczepanek K., Sumlet M., Kupiec M., Koczur-Szozda E., Steinhoff-Nowak M., Figula K., Kowalczyk T. (2011). Commercial enteral formulas and nutrition support teams improve the outcome of home enteral tube feeding. JPEN.

[15] British Association for Parenteral and Enteral Nutrition Introducing “MUST”. http://www.bapen.org.uk/screening-for-malnutrition/must/introducing-must.

[16] Department of Health (2001). Valuing People: A New Strategy for Learning Disability for the 21st Century.

[17] McLaughlin K.A. (2014). Home sweet home-can future enteral tube feeding for motor neurone disease begin in the community. CN.

[18] Care Quality Commission (2010). Essential Standards of Quality and Safety.

[19] Bermange J., Stewart K. (2013). Measuring clinical outcomes in a community home enteral feeding team. CN.

[20] Lämås K., Willman A., Lindholm L., Jacobsson C. (2009). Economic evaluation of nursing practices: A review of literature. Int. Nurs. Rev..

[21] Blomberg J., Lagergren J., Martin L., Mattsson F., Lagergren P. (2012). Complications after percutaneous endoscopic gastrostomy in a prospective study. Scan. J. Gastroenterol..

